# Effect of Digital Health Interventions on the Nutritional Status and Quality of Life of Patients With Colorectal Cancer: Systematic Review and Meta-Analysis of Randomized Controlled Trials

**DOI:** 10.2196/87866

**Published:** 2026-08-18

**Authors:** Xiujuan Feng, Jinna Wang, Huihui Shi, Rui Liang, Wenkai Zheng, Lijun Han, Kueh Yee Cheng, Ruhaya Hasan, Zhuoxin Wang, Ying Wei, Hafzan Yusoff

**Affiliations:** 1 Nutrition Programme, School of Health Sciences, Health Campus, Universiti Sains Malaysia, 4th FI., Kota Bharu, Kelantan, 16150, Malaysia, 60 17-9371499; 2 Department of Nursing, College of Nursing and Rehabilitation, Xi'an Jiaotong University City College, Xi 'an, Shaanxi Province, China; 3 Department of Traditional Chinese Medicine, Tangdu Hospital, The Fourth Military Medical University, Xi 'an, Shaanxi Province, China; 4 Department of Psychiatry, Shandong Mental Health Center, Jinan, Shandong Province, China; 5 Biostatistics and Research Methodology Unit, School of Medical Sciences, Health Campus, Universiti Sains Malaysia, Kota Bharu, Kelantan, Malaysia; 6 School of Dental Sciences, Health Campus, Universiti Sains Malaysia, Kota Bharu, Kelantan, Malaysia; 7 School of Economics and Management, Xi'an Jiaotong University City College, Xi 'an, Shaanxi Province, China

**Keywords:** digital health intervention, colorectal cancer, nutritional status, quality of life, randomized controlled trials

## Abstract

**Background:**

Digital health interventions may improve clinical outcomes in patients with colorectal cancer (CRC), but their impact on nutritional status (NS) and quality of life (QoL) remains unclear.

**Objective:**

This study systematically evaluated the impact of digital health interventions on the NS and QoL in patients with CRC.

**Methods:**

A systematic search was performed across PubMed, Embase, Cochrane, and Web of Science from inception to March 2026 to identify randomized controlled trials evaluating digital health interventions on NS or QoL in CRC. Two reviewers independently performed screening, data extraction, and risk of bias assessment (Cochrane Risk of Bias Assessment Tool [RoB 1.0]). Meta-analysis used a random-effects model to compute standardized mean differences (SMD) or mean differences (MD) with 95% CIs. Heterogeneity was assessed using *I*² and τ², and subgroup analyses (intervention duration, intervention method, and scale type) explored sources of heterogeneity. GRADE (Grading of Recommendations Assessment, Development, and Evaluation) was used for evidence quality.

**Results:**

Eleven RCTs from 7 countries, involving 1210 participants aged 37.7‐69.0 years. The meta-analysis shows that digital health interventions have no significant effect on improving NS (MD 2.23, 95% CI –10.50 to 14.97; *P*=.27), with high heterogeneity (*I*²=81.8%). The overall analysis of QoL shows that digital health interventions significantly improve the QoL of patients (SMD 0.67, 95% CI 0.14-1.21; *P*=.02), with a moderate effect size, but there is high heterogeneity (*I*²=92.9%; τ²=0.5146; 95% prediction intervals –1.04 to 2.38). Subgroup analysis indicates in terms of intervention duration, the effect is not significant in the group with ≤1 month, while it is significant in the groups with 1-3 months and >3 months, but there is no statistical significance in the differences among subgroups (*P*=.67); in terms of intervention methods, the group with guided intervention has a significant effect and no heterogeneity (SMD 1.16, 95% CI 1.05-1.28; *I*²=0%, τ²=0, *P*=.97), the effect of the unsupervised intervention group is zero, and the effect of the combined intervention group is unstable, with significant differences among subgroups (*P<*.001); in terms of scale types, different QoL measurement tools have certain effects on the effect size, but the differences among subgroups are not significant (*P*=.99).

**Conclusions:**

Digital health interventions have a moderate effect on improving the QoL in patients with CRC, especially when guided interventions are employed, the effect is clearer and more stable. This innovatively identifies “guided intervention models” as a key moderator of efficacy, contrasting with prior reviews that focused solely on technology. Our findings provide new evidence highlighting the importance of interpersonal support in digital health applications. In practice, digital tools should integrate professional medical guidance and supervision rather than being implemented in isolation, to more effectively improve health outcomes in patients with CRC.

## Introduction

Colorectal cancer (CRC), a malignancy of the colon or rectal mucosa, remains a major global health burden with rising incidence in both high-income and low- and middle-income regions [1]. Currently, standard treatments, including surgery with or without chemotherapy, radiotherapy, or targeted therapy, effectively control tumor progression but often induce significant physiological and psychological side effects [2]. Physiologically, surgical trauma and the side effects of chemoradiotherapy frequently induce gastrointestinal dysfunction, decreased appetite, and altered taste perception, leading to inadequate nutrient intake and malnutrition [3]. Psychologically, patients commonly experience emotional issues such as anxiety, depression, and sleep disorders [4]. Collectively, these factors severely impact patients’ nutritional status (NS) and overall quality of life (QoL). Research indicates that NS and QoL are not only key prognostic indicators for CRC but are also closely associated with treatment tolerance, complication rates, and long-term survival [5]. It is worth noting that multiple studies have indicated that patients with CRC have poorer NS and that their scores in various dimensions of QoL are generally lower than those of patients with other types of cancer [6]. Accordingly, strategies to enhance NS and QoL have become central to CRC care, prompting the exploration of innovative approaches such as digital health interventions.

The guidelines of the European Society for Clinical Nutrition and Metabolism (ESPEN) [7] emphasize the significance of integrating nutritional support throughout the entire cancer treatment trajectory. Conventional interventions to improve NS and QoL in patients with CRC have primarily relied on passive, standardized management within hospital settings. These typically include dietary guidance during hospitalization, oral nutritional supplementation, and discharge education based on printed materials [8]. While these conventional approaches serve a foundational role, their effectiveness is often constrained by inherent limitations, including strict confinement to hospital settings, lack of individualized plans, discontinuity postdischarge, and low patient engagement in self-management [9]. Digital health interventions, as an emerging health care model, offer a new pathway to overcome these challenges. The World Health Organization (WHO) defines digital health interventions as a systematic practice that combines digital technologies (eg, mobile devices, sensors, and computing devices) with health purposes, and uses data communication technologies to provide health services, conduct public health monitoring, or promote health education and management [10]. Leveraging diverse platforms such as smartphone apps, SMS text messaging, wearable devices, remote monitoring platforms, and online courses, this model capitalizes on its accessibility, convenience, and interactivity to enable continuous monitoring of patient NS, delivery of personalized dietary recommendations, real-time feedback, and enhanced physician-patient communication [11,12]. Recent studies have explored applying digital health technologies to improve nutritional outcomes and QoL for patients with cancer [13,14]. For instance, app-based nutrition management tools can help patients with CRC optimize their dietary patterns by recording food intake and providing immediate feedback [15]. Remote monitoring systems enable early identification and intervention for treatment-related malnutrition, potentially enhancing the QoL for patients with CRC [16]. These preliminary findings suggest that digital health interventions hold great promise for optimizing nutrition management and improving patients’ QoL.

At present, there is no consensus on the impact of digital health interventions on the NS and QoL of patients with CRC. First, the effect of digital health interventions on the QoL of patients with CRC is uncertain. In one meta-analysis of 10 studies, only 2 studies showed that mobile health (mHealth) intervention measures for CRC survivors improved QoL, while 3 studies did not find any significant differences [17]. A systematic review of RCTs on mHealth interventions in cancer survivors also found that, among the 11 studies that evaluated QoL, only 6 reported significant improvement, again demonstrating the inconsistency of the outcomes. Second, the evidence for NS outcomes is limited [18]. Currently, there are relatively few high-quality studies specifically focusing on nutritional outcomes. Although some studies have reported positive effects, such as a digital health intervention study for patients with gastrointestinal disorders with cancer showing that participants’ risk of malnutrition decreased by an average of 6.0 points after the intervention, the systematic evidence is still insufficient [19]. In summary, existing reviews have summarized the application of digital health interventions in patients with cancer, but most of them have included a wide range of cancer types and have not specifically analyzed CRC, and have paid less attention to QoL and NS. Therefore, this study aims to comprehensively evaluate the impact of digital health interventions on the NS and QoL of patients with CRC through systematic review and meta-analysis, in order to integrate existing evidence, clarify inconsistencies, and provide evidence-based support for clinical practice and future research.

## Methods

### Overview

The systematic review and meta-analysis were conducted in compliance with the PRISMA (Preferred Reporting Items for Systematic Reviews and Meta-Analyses) guidelines, and the PRISMA 2020 expanded checklist was used (Checklist 1) [20]. The protocol for this study was registered in the PROSPERO (International Prospective Register of Systematic Reviews); no amendments were made to the search protocol after the initial registration (CRD420251125990).

### Eligibility Criteria

The specific eligibility requirements of inclusion criteria followed the population, intervention, comparison, outcomes, and study format: (1) patients aged 18 years and above who have been diagnosed with CRC, colon cancer, or rectal cancer; (2) can be at any treatment stage (perioperative period, adjuvant chemotherapy period, maintenance treatment period, or rehabilitation period); (3) no restrictions on tumor stage, whether with stoma or metastasis; (4) intervention was delivered via a web-based remote digital health management system, such as mobile apps and AI. The intervention methods include “guided intervention (ie, feedback from health care providers or researchers during the intervention process)” and “unguided self-management intervention (fully automated feedback)” or a combination of both; (5) the comparison was between the control group that received only the usual care interventions and the intervention group that used the web-based remote digital health management system in addition to the usual care interventions; (6) the outcomes were the impacts of the interventions on overall or at least one type of relevant health-related outcomes (eg, NS and QoL); and (7) the study design was randomized controlled trials (RCTs).

The review excluded (1) studies with unclear diagnostic and efficacy criteria; (2) studies that were cohort studies, review articles, case reports, descriptive studies, opinion articles, gray literature, unpublished literature, or abstracts; (3) studies with incomplete or erroneous data that could not be merged; and (4) studies describing protocols, along with those focusing solely on the interface or internal structure of apps; and studies based on web pages or websites without associated apps.

### Information Sources

The literature search was initially designed according to our prospectively registered protocol, which restricted inclusion to English-language studies and searched 4 English databases. In the final manuscript, we adjusted the search strategy to enhance comprehensiveness and reduce language bias, and these modifications have been clearly reported as post hoc adjustments. The literature search for this systematic review was reported in accordance with the PRISMA-S (Preferred Reporting Items for Systematic Reviews and Meta-Analyses Literature Search Extension) statement [21]. A systematic search was conducted using subject terms combined with free terms, covering the following databases, including PubMed, Embase, Cochrane, and Web of Science. The search period began from the establishment of each database and lasted until March 2, 2026.

### Search Strategy

This search strategy for this systematic review was developed by drawing on and optimizing strategies from previous studies, and it did not undergo a specific peer review process. The search parameters were set to include only RCTs and peer-reviewed papers, with no restrictions on publication year or language, and no use of published search filters. To maximize the scope of the search, the snowball method was used for citation retrieval, that is, all the references of the included studies and their citations were checked. For missing outcome data in some studies, the relevant authors were contacted via email to supplement the original data that were not obtained through the initial search. This systematic review only used the above methods to retrieve literature. No supplementary search of research registration databases was conducted, nor was there targeted browsing of printed conference proceedings or government websites. The final search date for all databases was March 2, 2026. The detailed search strategy can be found in Multimedia Appendix 1.

### Selection Process and Data Collection Process

FXJ and SHH separately reviewed the identified papers to reduce potential errors and bias throughout the selection process. Initially, the authors screened the titles and abstracts of the potential papers against the set inclusion and exclusion criteria. Following this, the final selection of papers was made after thoroughly reading the complete manuscripts of the qualifying papers and their references. Any discrepancies were settled through discussions among the authors until a consensus was reached.

### Data Items

A structured data extraction form was used to gather the following details: the first author’s name; year of publication; country; study type; participant demographics (sample size and age); interventions for remote digital health management; intervention duration; involvement of health care professionals; measures to ensure participant compliance; and outcomes. The corresponding authors were contacted to clarify or obtain any information that was unclear or missing.

### Study Risk of Bias Assessment

FXJ and WJN performed the quality assessment independently. Any disagreements were resolved through discussion between the 2 researchers, and a third investigator independently reviewed the final decisions. The quality of the RCTs was evaluated by 2 researchers using the Cochrane Risk of Bias Assessment Tool (ROB1) for assessing risk of bias. This tool addresses 6 domains of bias, including selection bias, performance bias, detection bias, attrition bias, reporting bias, and other biases. The risk of bias was classified as high, low, or unclear, with reasons provided for each classification.

### Effect Measures

When all the indicators are measured using the same standard, we aggregate the continuous data through the mean difference (MD), such as NS; while when different measurement standards are used, we use the standardized mean difference (SMD) for aggregation, such as QoL.

### Synthesis Method

Considering the clinical heterogeneity in the population characteristics, specific contents of the intervention measures, and outcome measurement tools included in the study, we predetermined to adopt the random effects model as the main analytical framework [22]. This choice is based on conceptual assumptions rather than solely relying on the results of statistical heterogeneity tests. To improve the accuracy of effect size estimation in small samples or in the presence of heterogeneity, we used the Hartung-Knapp-Sidik-Jonkman (HKSJ) method to combine the random effects model and calculate the 95% CIs of the pooled effect size [22]. Effect sizes were interpreted using Cohen *d* criteria; values of 0.2, 0.5, and 0.8 represent small, medium, and large effects, respectively [23]. This systematic review uses restricted maximum likelihood estimation to estimate between-study variance, thereby more accurately reflecting the impact of true heterogeneity on effect estimates. To assess the heterogeneity among the studies more comprehensively, we used multiple indicators for quantification. For meta-analyses involving at least 3 studies, we reported the 95% CIs and 95% prediction intervals (PIs) to convey the range of effect sizes that new original studies might exhibit in different clinical scenarios in the future. For analyses with fewer than 3 studies, since the PIs might be too wide and lack information, they were not reported [24]. Meanwhile, we reported the *I*² statistic for comparison with previous studies but were cautious in interpretation, mainly by integrating τ² for a comprehensive judgment [22]. The standards recommended by the Cochrane Handbook are as follows: (0%‐40%) potentially insignificant heterogeneity; (30%‐60%) possibly moderate heterogeneity; (50%‐90%) possibly significant heterogeneity; and (75%‐100%) highly heterogeneous. It should be noted that these ranges are indicative only. The actual interpretation requires a comprehensive assessment considering the magnitude and direction of the effect size, the *P* value from the heterogeneity test (typically with *P*<.10 as the significance threshold), and the degree of overlap in the CIs presented in the forest plot [25]. To explore potential sources of heterogeneity, we conducted subgroup analyses based on intervention duration, intervention methods, and scale differences. To ensure the robustness of the results, a sensitivity analysis was conducted in this study (Multimedia Appendix 2). All statistical analyses were performed using R (version 4.5.2; R Foundation for Statistical Computing).

### Reporting Bias Assessment

By drawing funnel plots and combining with Egger regression intercept test, the influence of small sample effects on the results was evaluated. It should be emphasized that such methods mainly detect small sample effects rather than directly testing publication bias [26,27]. Small sample effects may be caused by various factors, including publication bias, selective reporting, true heterogeneity among studies, and methodological differences, etc. According to methodological standards, funnel plots only have reliable interpretative power when at least 10 studies are included; otherwise, their interpretability is relatively low [26,28]. We applied Egger regression intercept test to all outcome indicators. It should be noted that when the number of included studies is small, statistical power will significantly decrease, which may lead to the generation of false negative results [27]. The outcome indicators finally published in this review are completely consistent with the prespecified plan.

### Certainty Assessment

To appraise the certainty of evidence, we applied GRADE (Grading of Recommendations Assessment, Development, and Evaluation) guidance (GRADE Working Group) to the main outcomes (NS and QoL). RL and WKZ reviewers independently rated evidence across risk of bias, inconsistency, indirectness, imprecision, and publication bias, and resolved disagreements by consensus.

## Results

### Study Selection

**Figure 1.**
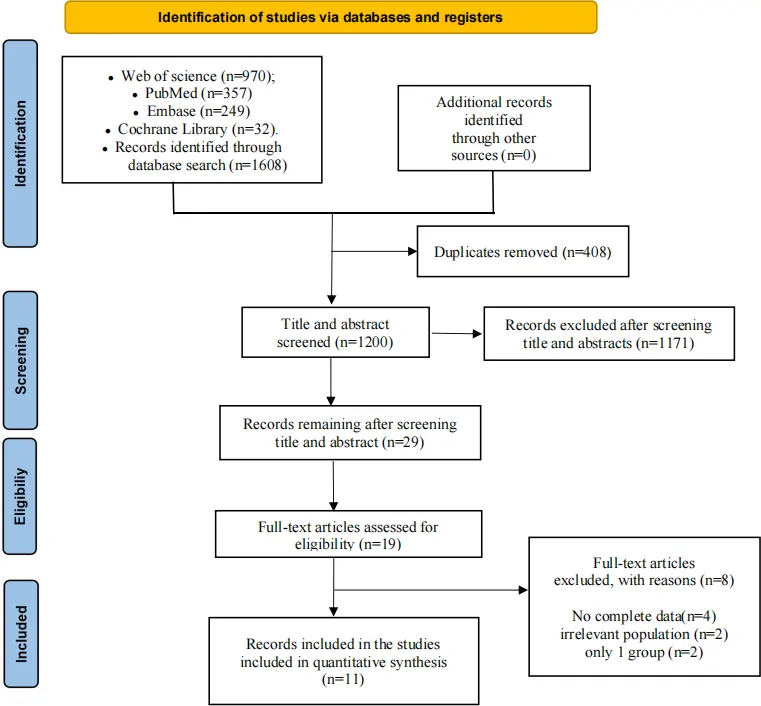
PRISMA (Preferred Reporting Items for Systematic Reviews and Meta-Analyses) flowchart detailing the screening process for randomized controlled trials from 4 databases, identifying 11 randomized controlled trials comparing digital health intervention to usual care intervention. RCT: randomized controlled trial.

The PRISMA flow diagram (Figure 1) illustrates the screening process and the criteria used for excluding papers. The initial search yielded 1608 publications. After deduplication using EndNote (Clarivate) software, 408 duplicate publications were excluded, leaving 1200 publications for title and abstract screening. Based on inclusion and exclusion criteria, 1171 publications that did not meet requirements were excluded, leaving 29 publications for full-text review. During the full-text review process, 8 studies were excluded due to incomplete data (n=4), irrelevant research subjects (n=2), or only one research group (n=2). Ultimately, 11 RCTs were included in the quantitative synthesis analysis [29-39].

### Study Characteristics

The 11 included studies originated from 7 countries, including Korea (n=3) [29,30,38], Thailand (n=1) [31], Spain (n=1) [32], the United States (n=1) [33], China (n=3) [34,36,37], the Netherlands (n=1) [35], and Vietnam (n=1) [39]. Publication dates spanned 2018-2026, with a total sample size of 1210 participants: 598 in the intervention group and 612 in the control group [29-39]. Participants' ages ranged from 37.7 to 69.0 years. Digital health interventions encompassed diverse formats, including lifestyle interventions via mHealth apps [29,32-35,37-39], smartphone-based exercise programs [30], and nutrition education programs based on mobile apps, online platforms, and social media [31,36], with intervention durations ranging from 2 to 48 weeks. Of the 11 studies, 8 [29-31,34-37,39] explicitly mentioned health care professionals (eg, coaches, nutritionists, nurses, and doctors) participating in the intervention process. Eight studies used specific strategies to enhance intervention adherence. Among them, 3 studies [29,33,35] used automated text messages or app push notifications sent by digital platforms according to preset schedules, with the content mostly consisting of standardized meal check-in reminders or general health tips. Four studies [30,34,36,37] involved follow-up calls or WeChat (Tencent Holdings Limited) messages from professional health care providers, along with face-to-face evaluations. These interactions typically featured personalized consultations, and researchers would provide targeted advice based on the specific data of the participants. One study [38] included both text messages sent by the digital platform and phone follow-ups by health care providers to enhance patient compliance. Regarding outcome measures, 10 studies [29,30,32-39] included QoL assessments, and 2 studies [31,36] conducted NS assessments. Detailed baseline information for included studies is presented in Table S1 in Multimedia Appendix 3.

### Risk of Bias in Studies

**Figure 2.**
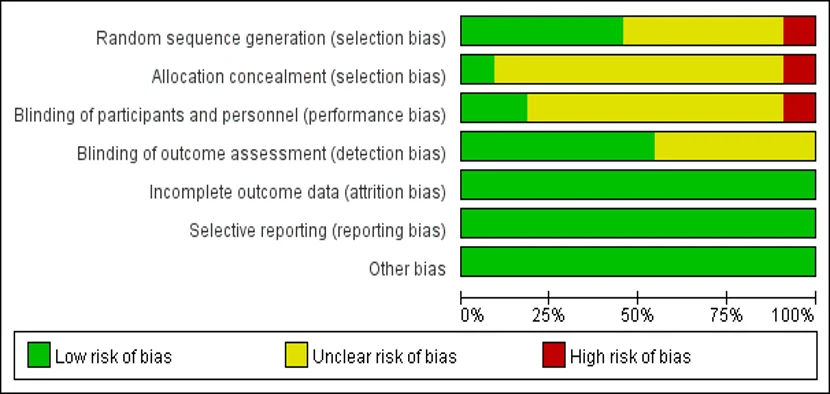
Risk of bias graph of randomized controlled trials.

**Figure 3.**
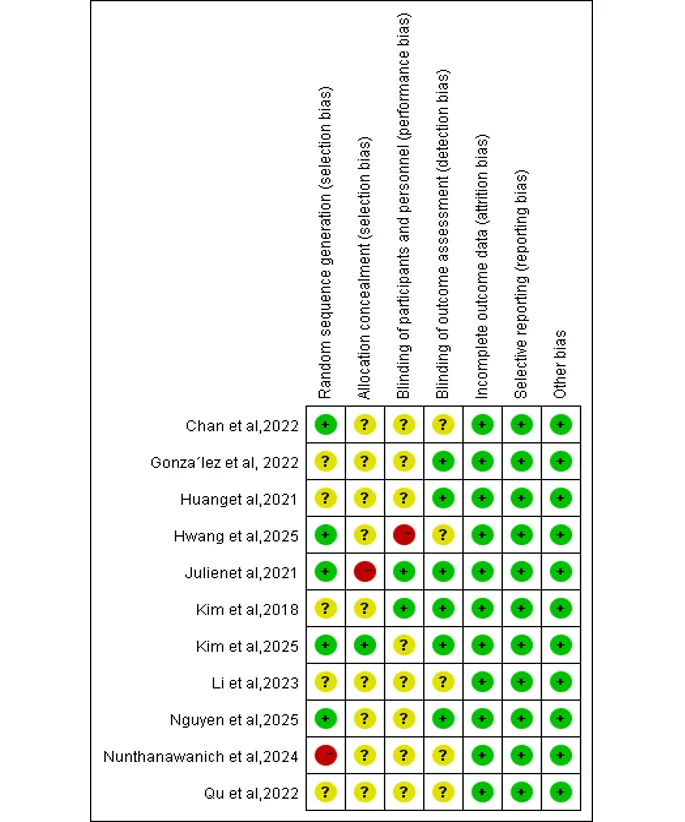
Risk of bias summary of randomized controlled trials. The review authors’ judgments about each risk of bias item are presented as percentages. The x-axis represents the percentage of studies that were found to have low (green), unclear (yellow), or high (red) risk of bias for each domain [29-39].

This study strictly adhered to the PRISMA guidelines, with 11 RCTs ultimately included. The Cochrane RoB 1.0 was adopted to evaluate the quality of the trials across 6 dimensions. Figures 2 and 3 illustrate the assessment of bias risk in RCTs. Among the 11 included studies, most had high quality in terms of attrition bias, reporting bias, and other biases, all rated as low risk [29,30,32-39]. However, there were many methodological issues in terms of selection bias and implementation bias. Specifically, in the generation of random sequences, 1 (9%) study [31] was rated as high risk due to the use of non-random methods such as order of admission, 5 (45%) studies [32,34,36-38] were rated as unclear because they only mentioned randomization but did not describe the specific generation method; in the aspect of allocation concealment, 9 (82%) studies [30-34,36-39] were rated as unclear because they did not report the implementation details of allocation concealment (eg, sealed envelopes or central randomization); in the aspect of participant and personnel blinding, 1 (9%) study [30] was rated as high risk due to the lack of implementation of blinding or the blinding being compromised, and 8 (73%) studies [29,31-34,36,37,39] were classified as unclear due to the incomplete description of the blinding situation in the reports; in the aspect of outcome assessment blinding, 5 (45%) studies [30,31,33,36,37] were rated as unclear because the reporting did not clearly describe the situation of outcome assessors knowing the group allocation. The dropout rate in this study was low and the groups were well balanced, indicating that the risk of dropout bias was within an acceptable range.

### Results of Individual Studies

In terms of management intervention measures, 8 trials [29-31,34-37,39] involved the participation of medical professionals in using digital health tools, while the remaining 3 [32,33,38] did not provide specific information (Table S1 in Multimedia Appendix 3). The main roles of medical professionals include developing personalized intervention plans [29,37], providing nutritional guidance and exercise training [29,31], guiding device use [30,37], reviewing health data [29,35], and conducting subsequent question answering and plan adjustments [34,36]. In all studies, a total of 11 different digital health tools were used, covering 6 mHealth apps, 2 web programs, 2 smart devices, and 1 tool that combines a web page with WeChat. These tools were mainly applied in 2 key areas of CRC management, including self-management [30,31,33,36-39] and rehabilitation support [29,32,34,35]. Self-management platforms typically have functions such as health knowledge dissemination, data recording, goal setting, and question answering; rehabilitation support platforms focus on tracking postoperative recovery data, monitoring physical activities, and enabling real-time viewing and intervention by the medical team on the rehabilitation status. Some tools require the use of smart devices (eg, wristbands) and can display health information in real time through the connection to the device screen. An overview of the characteristics of digital health tools is shown in Table S2 (Multimedia Appendix 3).

### Results of Syntheses

#### NS

**Figure 4.**
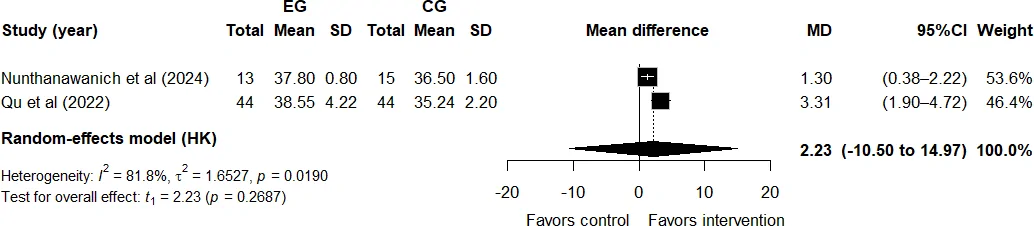
Forest plot of NS assessed after the intervention [31,36]. CG: control group; EG: experimental group; HK: Hartung-Knapp (method); MD: mean difference.

Two studies [31,36] examined the effect of digital health interventions on patient NS, using albumin levels as the primary outcome measure. Meta-analysis results (Figure 4) showed that, using a random-effects model to pool data, there was no statistically significant difference in improving NS between the intervention group and the control group (MD 2.23, 95% CI –10.50 to 14.97; *P*=.27). The heterogeneity test indicates that there is a high degree of heterogeneity among these studies (*I*²=81.8%; τ²=1.6527; *P*=.02). Due to the limited number of included studies, the results should be interpreted with caution.

#### QoL Outcomes

**Figure 5.**
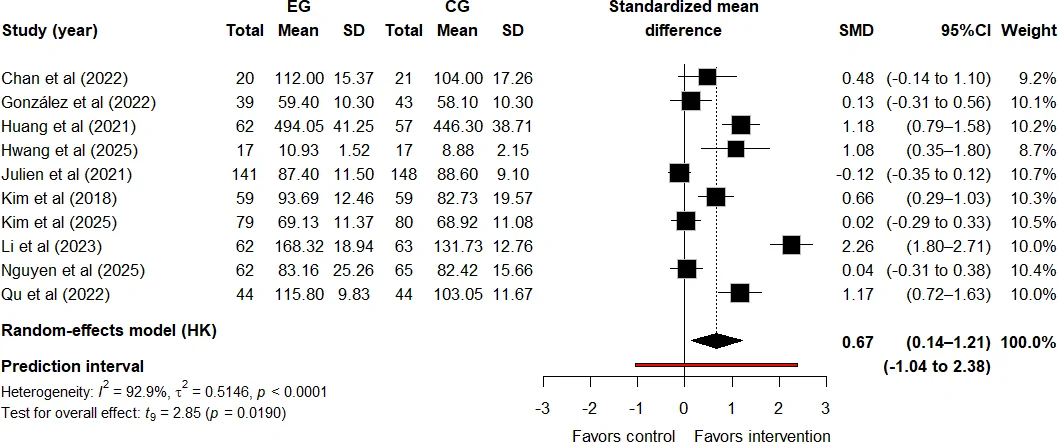
Forest plot of quality of life (QoL) after intervention assessment [29,30,32-39]. CG: control group; EG: experimental group; HK: Hartung-Knapp (method); SMD: standardized mean difference.

A meta-analysis of 10 studies [29,30,32-39] examining digital health interventions on patients’ QoL revealed (Figure 5) that the QoL score of the intervention group was higher than that of the control group (SMD 0.67, 95% CI 0.14-1.21; *P*=.02). SMD of 0.67 for QoL represents a moderate effect size. However, it also had a high degree of heterogeneity (*I*²=92.9%; τ²=0.5146; *P*<.0001), and the 95% PI is –1.04 to 2.38, indicating that the intervention effect may fluctuate significantly in clinical apps.

**Figure 6.**
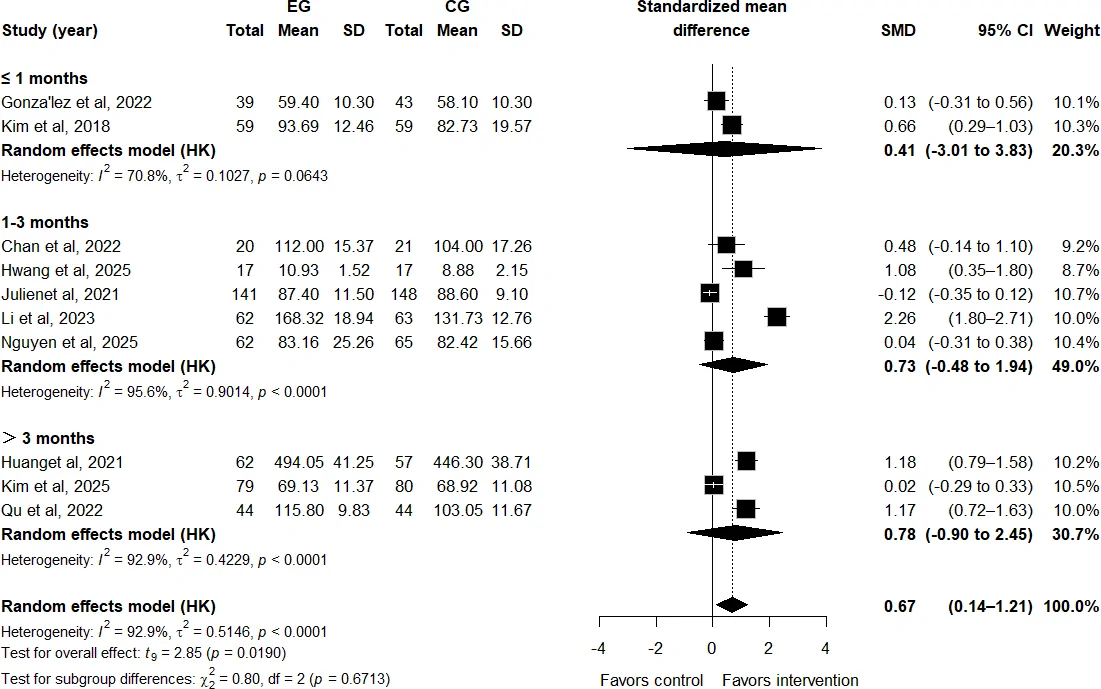
The subgroup analysis results regarding the impact of the duration of digital health intervention measures on QoL [29,30,32-39]. CG: control group; EG: experimental group; HK: Hartung-Knapp (method); SMD: standardized mean difference.

Given the high heterogeneity in the meta-analysis of the overall QoL, further subgroup analyses were conducted. A subgroup analysis based on the duration of intervention showed (Figure 6) that the digital health intervention had no statistically significant effect on improving QoL when the intervention duration was ≤1-month (SMD 0.41, 95% CI –3.01 to 3.83; *I*²=70.8%; τ²=0.1027; *P*=.06). However, when the intervention duration was in the 1‐3 month group (SMD 0.73, 95% CI –0.48 to 1.94; *I*²=95.6%; τ²=0.9014; *P*<.001) and >3-month group (SMD 0.78, 95% CI –0.90 to 2.45; *I*²=92.9%; τ²=0.4229; *P*<.001), it had a statistically significant effect on improving QoL. Although the point estimate indicated a slight increase in the effect size as the intervention duration increased, the subgroup difference test results showed that this difference was not statistically significant (*χ*²_2_=0.80; *P*=.67). This subgroup analysis was a post hoc stratified analysis. This lack of significant difference is merely an exploratory observation pattern and does not constitute conclusive evidence that the intervention duration has no impact on the improvement of QoL.

**Figure 7.**
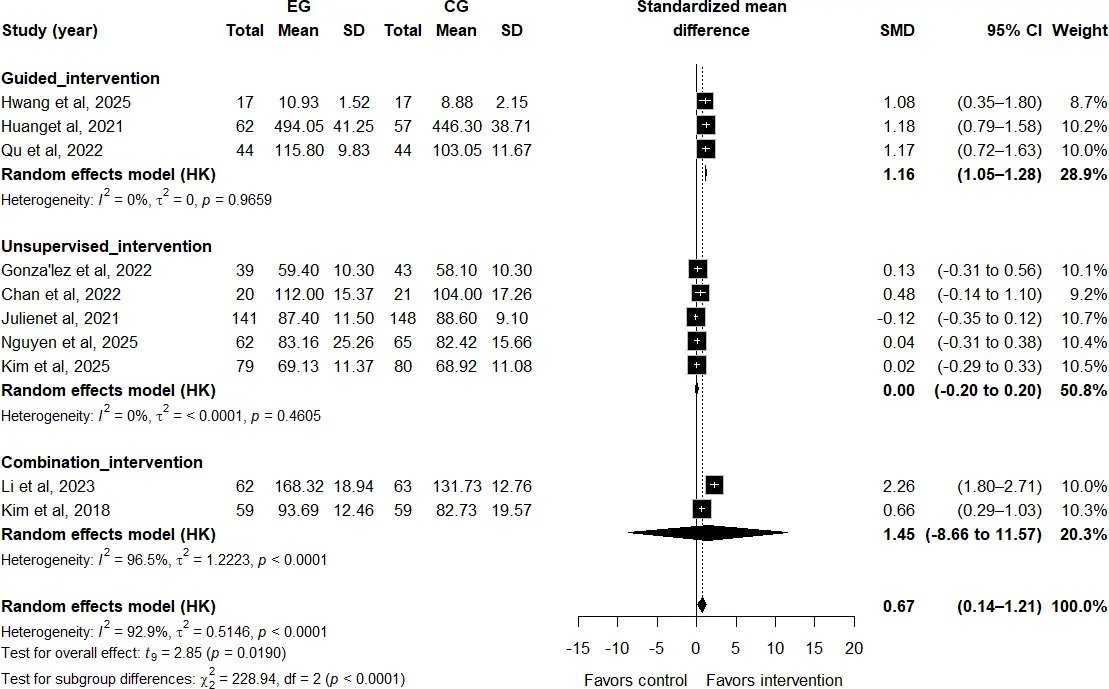
The subgroup analysis results regarding the impact of the methods of digital health intervention on quality of life (QoL) [29,30,32-39]. CG: control group; EG: experimental group; HK: Hartung-Knapp (method); SMD: standardized mean difference.

The subgroup analysis based on the intervention methods (Figure 7) showed that the digital health intervention had a significant positive impact on QoL in the guided intervention group (SMD 1.16, 95% CI 1.05-1.28; *I*²=0%; τ²=0; *P*=.97). However, in the unsupervised intervention group (SMD 0.00, 95% CI –0.20 to 0.20; *I*²=0%; τ² <0.0001; *P*=.46) and the combined intervention group (SMD 1.45, 95% CI –8.66 to 11.57; *I*²=96.5%; τ²=1.2223; *P*<.0001). The subgroup difference test showed that the differences in effect between different intervention methods were statistically significant (*χ*²_2_=228.94; *P*<.0001), suggesting that the intervention method is an important factor influencing QoL.

**Figure 8.**
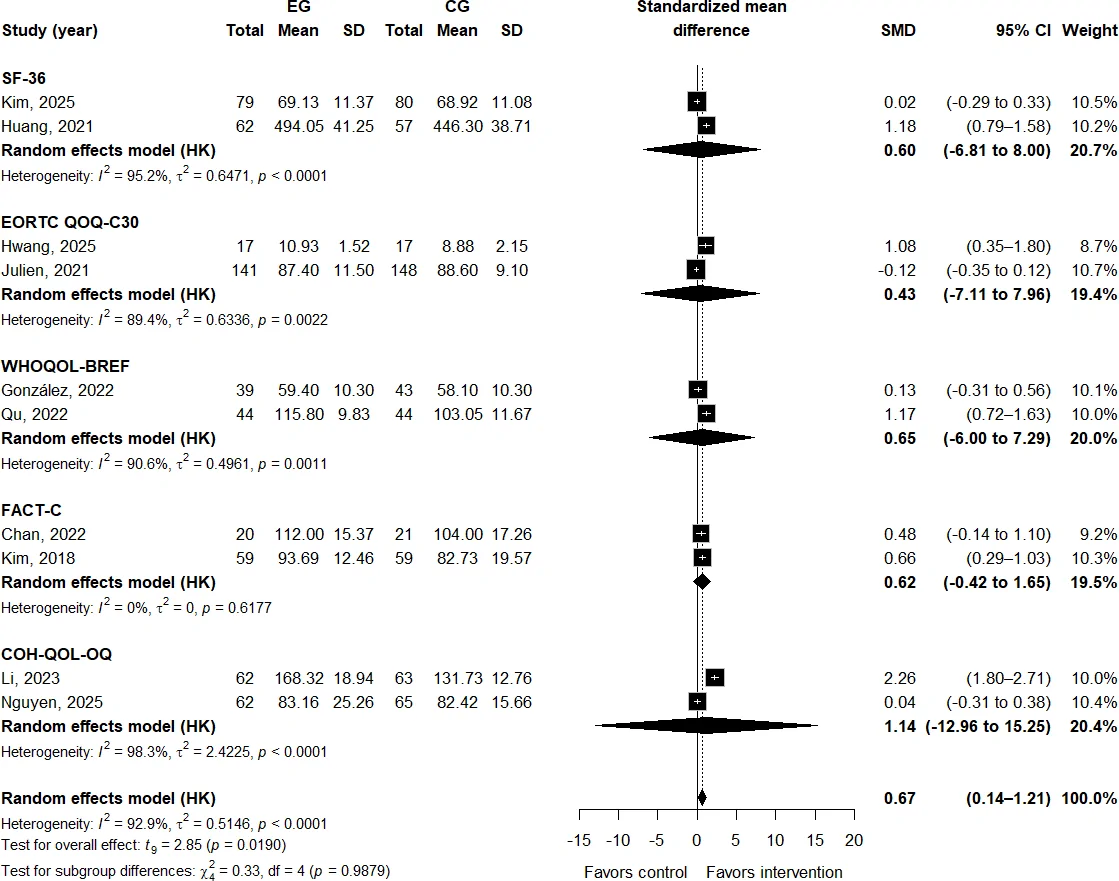
The subgroup analysis results regarding the scale differences on quality of life (QoL) [29,30,32-39]. CG: control group; EG: experimental group; HK: Hartung-Knapp (method); SMD: standardized mean difference.

The subgroup analysis based on the types of QoL scales showed (Figure 8) that the digital health intervention demonstrated a larger effect size in the FACT-C (Functional Assessment of Cancer Therapy–Colorectal) scale (SMD 0.62, 95% CI –0.42 to 1.65; *I*²=0%; τ²=0; *P*=.62) and the Hope-Quality of Life-Ostomy Questionnaire (COH-QoL-OQ) scale (SMD 1.14, 95% CI –12.96 to 15.25; *I*²=98.3%; τ²=2.4225; *P*<.001), but the latter had extremely high heterogeneity and the results were unstable. In the 36-Item Short Form Health Survey (SF-36) scale (SMD 0.60, 95% CI –6.81 to 8.00; *I*²=95.2%; τ²=0.6471; *P*<.001), the European Organization for Research and Treatment of Cancer—Quality of Life Core 30 (EORTC QOG-C30) scale (SMD 0.43, 95% CI –7.11 to 7.96; *I*²=89.4%; τ²=0.6336; *P*=.002), and the World Health Organization Quality of Life-Brief version (WHOQoL-BREF) scale (SMD 0.65, 95% CI –6.00 to 7.29; *I*²=90.6%; τ²=0.4961; *P*=.0011), the effect size was moderate, but the CIs were wide and the heterogeneity was high. The subgroup difference test results showed that the effect differences between different scale types were not significant (*χ*²_4_=0.33; *P*=.99).

### Reporting Biases

**Figure 9.**
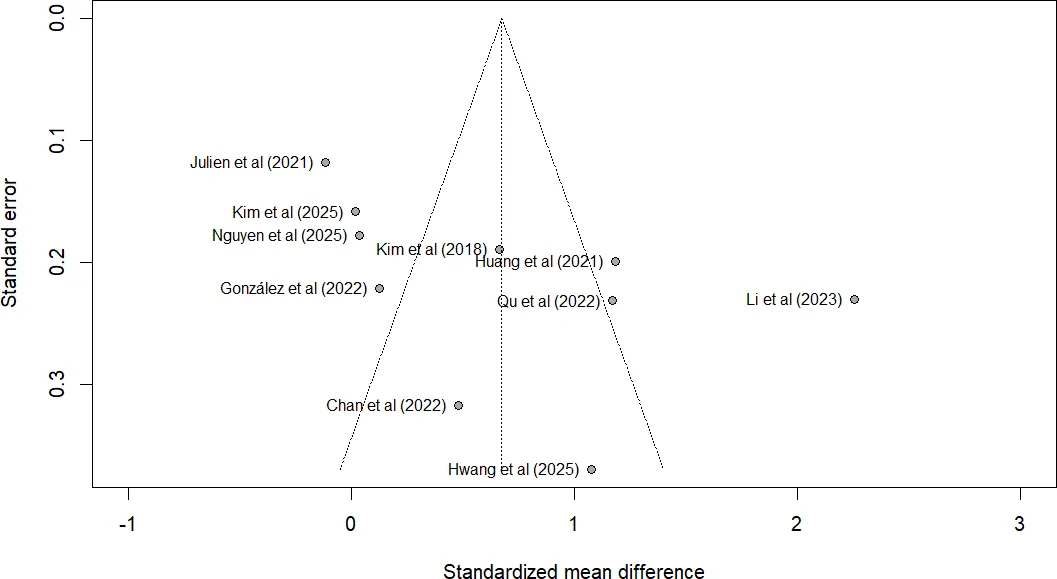
Funnel plot of quality of life (QoL).

To assess the impact of small studies, we combined visual inspection of the funnel plot with Egger regression intercept test in accordance with methodological guidelines [40]. We constructed a funnel plot for QoL (Figure 9) to visually explore potential asymmetry and did not detect a significant influence of small studies (z=1.29; *P*=.20). However, because only 10 studies were included, the symmetry of the funnel plot or the nonsignificant result of the Egger test cannot rule out the presence of publication bias. Moreover, if asymmetry is observed in the funnel plot, it may be due to heterogeneity or methodological flaws rather than solely publication bias [40]. Therefore, the current results do not rule out the possibility of publication bias or selective reporting bias, as the number of included studies is small, and these biases may not have been detected yet.

### Certainty of Evidence

Based on the GRADE evidence grading method, the quality of evidence regarding the effect of digital health interventions on improving NS and QoL in patients with CRC was evaluated. For the outcome of NS, 2 RCTs were included [31,36]. Due to serious risk of bias (inadequate reporting of randomization and blinding, reliance on subjective scales for outcome measurement), significant inconsistency (large discrepancies in effect estimates across studies), and serious imprecision (the 95% CI crossing the line of no effect and a small sample size), the overall quality of evidence was rated as low, indicating that the true effect is highly uncertain and subsequent research is very likely to change the current conclusion. For the outcome of QoL, 10 RCTs were included [29,30,32-39]. The risk of bias was acceptable, and no downgrading was applied for indirectness or imprecision. However, some heterogeneity existed across studies (variations in effect sizes due to differences in intervention methods, duration, and measurement tools), leading to a one-level downgrade for inconsistency. The final evidence quality was rated as moderate, with the pooled effect showing that digital health interventions can moderately improve patients’ QoL. This suggests that the true effect is likely to be close to the estimate, though some possibility remains that future studies may modify the finding. Results are summarized in Table 1.

**Table 1.** Grading of Recommendations Assessment, Development, and Evaluation (GRADE) assessment.

Certainty assessment							Number of patients		Effect		Certainty^a^	Importance
Number of studies	Study design	Risk of bias	Inconsistency	Indirectness	Imprecision	Other considerations	Digital health interventions	Usual care intervention	Relative (95% CI)	Absolute (95% CI)		
Nutritional Status												
2	Randomized trials	Serious^b^	Serious^c^	Not serious	Serious^d^	None	57	59	—	MD^e^ 2.23 SD lower (10.50 lower to 14.97 lower)	⨁◯◯◯^f^ Very low	Critical
Quality of Life												
10	Randomized trials	Not serious	Serious^b^	Not serious	Not serious	None	585	597	—	SMD^g^ 0.67 SD higher (0.14 higher to 1.21 higher)	⨁⨁⨁◯^h^ Moderate	Critical

a For both outcomes, higher scores indicate better status. ”Lower” and “higher” compare the intervention group with usual care; positive effects favor intervention, negative effects favor usual care.

b The study did not clearly report the randomization method or blinding, and the trial was judged to have an unclear risk of bias. The outcome measures used subjective scales, which may lead to an overestimation of the treatment effect. Therefore, due to concerns about the risk of bias, the certainty of the evidence was downgraded by one level.

c The prediction interval is wide, reflecting both the possibility of clinically meaningful benefits and the inability to rule out no effect or potential risks. The degree of heterogeneity among studies is high, indicating significant discrepancies in the estimated effects across different studies. Therefore, due to the inconsistency of the evidence, the certainty of the relevant evidence is downgraded by one level.

d The 95% CI is relatively wide and crosses the line of no effect, indicating that there is still considerable uncertainty about the true effect size. Based on this imprecise result, the corresponding level of evidence is downgraded by one level.

e MD: mean difference.

f ⨁◯◯◯ indicates very low certainty of evidence (only one level achieved).

g SMD: standardized mean difference.

h ⨁⨁⨁◯ indicates moderate certainty of evidence (three levels achieved).

## Discussion

### Principal Findings

This study conducted a meta-analysis on the effects of digital health interventions on the NS and QoL of patients with CRC. Regarding NS, based on 2 studies using albumin as an indicator, the analysis showed that digital health interventions did not significantly improve the NS of patients with CRC. The pooled effect size was not statistically significant, and there was high heterogeneity between the studies, so the results should be interpreted with caution. Regarding QoL, a comprehensive analysis of 10 studies indicated that digital health interventions significantly improved the QoL of patients with CRC overall, with a moderate effect size. However, there was high heterogeneity among the studies, and the prediction interval was wide, suggesting that the intervention effect may fluctuate considerably across different populations. To explore the sources of heterogeneity, further subgroup analyses were conducted; the intervention duration subgroup showed that short-term intervention had no significant effect, while medium-term and long-term interventions had significant effects, but the differences among the subgroups were not statistically significant. The intervention method subgroup revealed that supervised interventions had a stable and significant effect, unsupervised interventions had a minimal effect, and combined interventions had an unstable effect. The difference between these subgroups was significant, indicating that the type of intervention is an important effect modifier. The QoL scale subgroup showed that different measurement tools had some influence on the effect size, but the difference between subgroups was not significant. In conclusion, digital health interventions have positive potential for improving patients’ QoL, especially in supervised intervention models where the effect is more pronounced. However, their effect on improving NS remains unclear, and more high-quality research is needed for further verification in the future.

### Grading of Quality of Evidence

The quality of the included literature was generally high, with a low risk of bias. However, most studies did not mention the use of assessor blinding in outcome measurement [30,31,33,36,37]. Future research should aim to improve the design related to blinding. In the GRADE assessment, the evidence level for NS was rated as low [31,36], and the evidence level for QoL was rated as moderate [29,30,32-39]. Even after conducting sensitivity analyses, heterogeneity persisted across studies. The risk of bias for all outcome measures was downgraded due to issues with allocation concealment and inadequate implementation of blinding methods [29,30,36,37,39]. Consequently, researchers should prioritize standardizing experimental designs and meticulously executing these processes in future studies.

### Effects of Interventions on the NS and QoL

#### NS Outcomes

This meta-analysis of the study preliminarily explored the impact of digital health intervention measures on the NS in patients with CRC [31,36]. The combined results showed that albumin of the intervention group was improved compared to the control group, but did not reach a statistically significant level. This result may be influenced by several factors. First, insufficient statistical power might be the main reason for the negative result. This analysis only included 2 studies, with a limited total sample size, and the CI of the combined effect was wide, crossing the null line, indicating that the precision of the effect estimation was low [41]. In this case, even if there were real clinical benefits, it would be difficult to detect significant differences statistically.

Furthermore, there was high heterogeneity among the studies, suggesting that the results of the 2 studies were not consistent. Qu et al [36] showed that the NS of the intervention group improved significantly, while Nunnathanawinich et al [31] showed an improvement trend but with a smaller effect. This heterogeneity might stem from differences in the baseline characteristics of the study populations, intervention schemes, or the measurement time points of the outcome indicators. For example, Qu et al [36] had a larger sample size and might have included patients with poorer baseline NS, thus observing a more significant improvement effect. It is worth noting that the effect directions of the 2 studies [31,36] were consistent, both pointing to the potential benefits of the intervention on NS. This trend suggests that this intervention measure may have a clinically significant nutritional improvement effect, but larger sample sizes and higher-quality studies are needed to verify it.

#### QoL Outcomes

This study conducted a meta-analysis of multiple RCTs examining the impact of digital health interventions on patients’ QoL. The results showed that digital health interventions, overall, significantly improved patients’ QoL, with a moderate effect size [29,30,32-39]. This effect may be associated with the following 3 factors. First, by using tools such as mobile apps and remote monitoring systems, these interventions dynamically track patients’ dietary intake, physical activity, and symptom changes [29,32-35,37-39]. This enables timely adjustments to intervention plans, thereby helping to reduce health risks and minimize the impact of disease fluctuations on QoL [42,43]. Second, tailored nutritional advice and rehabilitation exercise plans are developed based on patients’ specific disease stages and treatment phases, closely aligning with their individual needs [30,34,36-38]. This allows patients to better manage their treatment process with improved physical condition, indirectly enhancing their QoL [44]. Third, simplifying the communication between patients and caregivers has overcome the barriers of time and space, enabling patients to quickly obtain guidance and psychological support from medical professionals [30,34,36]. Such access can alleviate anxiety, improve treatment adherence, and consequently enhance QoL by strengthening psychological well-being and treatment compliance [45-47].

Although digital interventions can improve QoL, 3 [29,35,39] out of the 11 included studies did not find a significant impact on QoL. This may be attributed to the relatively high proportion of older adult participants in these 3 studies. The physiological and psychological characteristics of the older adult population may, to some extent, diminish the impact of digital interventions on QoL [48]. The effectiveness of digital health interventions heavily depends on users’ technological acceptance and operational adherence [49]. Some older adult individuals may face challenges such as limited digital literacy or declining sensory functions (eg, vision impairment and reduced hand dexterity), which could restrict the effectiveness of the interventions [50]. Second, older adult patients often have complex clinical backgrounds, with multiple chronic conditions coexisting. The use of multiple medications or concurrent treatment regimens may interact, potentially masking or reducing the impact of digital interventions on overall QoL [51]. Therefore, future research should conduct stratified analyses based on different age groups and develop more user-friendly digital intervention programs.

To explore the potential sources of heterogeneity, this study conducted subgroup analyses based on intervention duration, intervention method, and scale type. In terms of the duration of the intervention, the subgroup analysis showed that the short-term intervention did not demonstrate statistically significant effects, while the medium-term and long-term interventions both showed significant improvement effects [29,30,32-39]. Although the point estimates increased slightly with the extension of the intervention time, the subgroup difference test did not reach the statistical significance level, suggesting that the duration of the intervention may not be the core factor independently explaining the heterogeneity, but this trend still deserves attention. The longer the digital health intervention lasts, the higher the QoL is, which is consistent with the research results of Gonzalez et al [32]. Second, the short-term intervention often overlaps with the key treatment stages of patients, during which physical discomfort (eg, nausea, vomiting, and wound pain) may mask the benefits of the intervention measures [35]. In contrast, long-term intervention can produce cumulative effects through continuous behavioral intervention and psychological support, leading to gradual improvements in QoL [52]. This aligns with the outcomes of Huang et al [34], who provided 6 months of online continuous care for patients with an ostomy, and Qu et al [36], who conducted a 12-week nutritional intervention. Therefore, clinical practice recommends setting intervention cycles at one month or longer to maximize benefits.

In terms of intervention methods, the guided intervention group showed the most stable and significant effects, suggesting that digital health interventions with professional guidance may be more effective in improving patients’ QoL [30,34,36]. However, the unguided intervention group did not show significant effects, indicating that relying solely on patients’ self-use of digital interventions may be difficult to produce substantial improvements in QoL [29,32,33,35,39]. This finding may be related to the fact that guided interventions can enhance the compliance of patients with CRC. The main measures adopted in this study include combining digital therapy with regular manual supervision (eg, telephone follow-up and video guidance) to improve long-term compliance [29,30,34-36,38]; incorporating game elements (eg, points, badges, and leaderboards), push notifications, and simplified user interfaces to enhance participation by reducing cognitive load and increasing interest [29,35,37]; developing intelligent adaptive systems to monitor behavioral data in real time through wearable devices and trigger personalized interventions, and establishing predictive warning models to intervene in high-risk users in advance [33]; implementing dynamic personalized strategies to adjust the difficulty and content of the intervention according to the user’s progress and feedback, and using micro-learning modules to reduce the burden of a single task [36]. The collaborative application of these measures can significantly improve compliance, thereby improving the QoL of patients with CRC, providing an evidence-based basis for the implementation of future digital health interventions.

In terms of the type of QoL scales, the subgroup analysis showed that the QoL measurement tools had a certain impact on the combined effect size, but the subgroup differences were not significant [29,30,32-39]. SF-36, as a general indicator, can conduct extensive comparisons of physical and mental health conditions [53]. EORTC QLQ-C30 and FACT-C are specific assessment tools for cancer, and their functional indicators and core symptoms such as fatigue and pain are relatively sensitive [54,55]. WHOQoL-BREF mainly emphasizes subjective well-being and environmental factors [56]. COH-QoL-OQ mainly assesses the QoL of patients with CRC ostomy, mainly evaluating psychological, social, and mental factors [57]. The WHOQoL-BREF scale focuses on the psychological aspect, the COH-QoL-OQ scale focuses on ostomy care, the SF-36, EORTC QLQ-C30, and FACT-C scales focus on overall health status, and each scale has differences in scoring standards and sensitivity, which may lead to higher heterogeneity in corresponding subgroups [53-57]. Although the subgroup analysis showed that the type of scale did not significantly regulate the intervention effect, the instability of the measurement results of different scales suggests that in future research, when choosing measurement tools, priority should be given to tools with strong tumor specificity and lower heterogeneity in similar studies. It is recommended to use EORTC QLQ-C30 as the core outcome indicator to ensure the comparability of the study and the robustness of the results [58]. When necessary, FACT-C and other scales can be used in combination to capture changes in different dimensions of QoL [59], but it should avoid using scales with poor stability to avoid exaggerating or masking the true intervention effect.

### Innovation and Limitations

The innovation of this study mainly lies in the following aspects: first, it focuses on the application of digital health intervention in the specific group of patients with CRC and examines its dual effects on NS and QoL, filling the gap in existing research where insufficient attention was paid to nutritional outcomes. Second, through detailed subgroup analysis, the study first clearly indicates that the intervention method (especially the guidance-based intervention) is the key regulatory variable leading to differences in effects, and reveals that the guidance-based intervention can stably improve the QoL in the case of zero heterogeneity, providing precise directional guidance for the design of subsequent intervention plans.

This study also has certain limitations. First, although 11 RCTs were included, the overall sample size is relatively limited, and there are significant differences in intervention duration and outcome measurement tools among the studies, resulting in high heterogeneity in the combined analysis, which to some extent weakens the robustness of the conclusion. Second, in the evaluation of NS, the study only uses the albumin level as the core indicator, which is relatively single and fails to comprehensively reflect the complex NS of patients (weight changes, muscle mass, dietary intake, etc), and the high heterogeneity of this outcome suggests that potential confounding factors may not have been adequately controlled. Third, the search strategy of this study is limited to published literature. It does not systematically search for gray literature and unpublished data from clinical trial registration platforms. This limitation may lead to potential publication bias, that is, some negative or unpublished results may be missed. Future research should adopt larger-scale and longer-term investigation methods and use standardized assessment standards to implement these standards in different age groups to further verify these findings.

### Conclusions

This systematic review indicates that digital health interventions show certain potential in improving the QoL of patients with CRC, with a combined effect size at a medium level. However, the interpretation of this result should be done with caution. First, the quality of evidence is limited by the high heterogeneity of the included studies, with a wide prediction interval, suggesting that the effect may not be significant or even have a negative effect in specific populations or intervention contexts. The stability and universality of the effect still need to be verified. Second, subgroup analysis reveals that the improvement in intervention effect may be related to specific design features, especially the guidance-based interactive mode and an intervention period of at least one month, indicating that not all digital interventions can produce equivalent benefits. The self-monitoring mode relying solely on technical means has limited effect. Finally, there is still a lack of sufficient evidence for the effectiveness of digital interventions in improving objective nutritional indicators (eg, albumin levels) of patients. Therefore, future research should not stop at verifying the overall effectiveness but should further focus on exploring the response differences of patients with different characteristics (eg, age, digital literacy, and disease stage) and develop evidence-based, more adaptable stratified intervention strategies in order to achieve truly precise health management in the future.

## Supplementary material

10.2196/87866 Multimedia Appendix 1 Search strategy.

10.2196/87866 Multimedia Appendix 2 Results of sensitivity analyses.

10.2196/87866 Multimedia Appendix 3 Detailed baseline information for included studies and overview of digital health tools characteristics.

10.2196/87866 Checklist 1 PRISMA checklist.

## Abbreviations

COH-QOL-OQ: Hope-Quality of life-Ostomy Questionnaire
CRC: colorectal cancer
EORTC QLQ-C30: European Organization for Research and Treatment of Cancer—Quality of Life Core 30
ESPEN: European Society for Clinical Nutrition and Metabolism
FACT-C: Functional Assessment of Cancer Therapy–Colorectal
GRADE: Grading of Recommendations Assessment, Development, and Evaluation
HKSJ: Hartung-Knapp-Sidik-Jonkman
MD: mean difference
mHealth: mobile health
NS: nutritional status
PI: prediction interval
PRISMA: Preferred Reporting Items for Systematic Reviews and Meta-Analyses
PRISMA-S: Preferred Reporting Items for Systematic Reviews and Meta-Analyses Literature Search Extension
PROSPERO: International Prospective Register of Systematic Reviews
QoL: quality of life
RCT: randomized controlled trial
ROB1: Risk of Bias Assessment Tool
SF-36: 36-Item Short Form Health Survey
SMD: standardized mean difference
WHO: World Health Organization
WHOQOL-BREF: World Health Organization Quality of Life-Brief

## References

[1] Bray F, Laversanne M, Sung H (2024). Global cancer statistics 2022: GLOBOCAN estimates of incidence and mortality worldwide for 36 cancers in 185 countries. CA Cancer J Clin.

[2] Ott JM, Gassenmaier V, Bitzer M, Schürch CM, Heitmann JS, Hagelstein I (2025). B7-H3: a consistent marker in metastatic colorectal cancer with potential for targeted treatment. Pathol Oncol Res.

[3] Bao LC, He Y (2025). Application of precision nutrition intervention based on patient subjective global assessment in the care of colorectal cancer patients. CJGP.

[4] Wang Q, Wang F, Zhang S, Liu C, Feng Y, Chen J (2023). Effects of a mindfulness-based interventions on stress, burnout in nurses: a systematic review and meta-analysis. Front Psychiatry.

[5] Hu D, Yu J, Feng J, Liu P, Chen JM, Zhang HL (2025). Comparison and trend analysis of cancer incidence in China and globally in 2022. World J Clin Oncol.

[6] Lindberg-Scharf P, Emmert M, Koller M (2025). Preference-oriented quality of life monitoring and linkage with clinical registry data: study protocol of a randomised clinical trial in patients with lung cancer (LePaLuMo Study). Trials.

[7] Muscaritoli M, Arends J, Bachmann P (2021). ESPEN practical guideline: clinical nutrition in cancer. Clin Nutr.

[8] Dingemans AM, van Walree N, Schramel F (2023). High protein oral nutritional supplements enable the majority of cancer patients to meet protein intake recommendations during systemic anti-cancer treatment: a randomised controlled parallel-group study. Nutrients.

[9] Roberts AL, Potts HW, Koutoukidis DA, Smith L, Fisher A (2019). Breast, prostate, and colorectal cancer survivors’ experiences of using publicly available physical activity mobile apps: qualitative study. JMIR Mhealth Uhealth.

[10] Kipruto H, Muneene D, Droti B (2022). Use of digital health interventions in Sub-Saharan Africa for health systems strengthening over the last 10 years: a scoping review protocol. Front Digit Health.

[11] Merino M, Del Barrio J, Nuño R, Errea M (2024). Value-based digital health: a systematic literature review of the value elements of digital health care. Digit Health.

[12] Gentili A, Failla G, Melnyk A (2022). The cost-effectiveness of digital health interventions: a systematic review of the literature. Front Public Health.

[13] Bao Y, Chen S, Jiang R (2020). The physical activity of colorectal cancer survivors during chemotherapy: based on the theory of planned behavior. Support Care Cancer.

[14] Savage LC, Soto-Cossio LE, Minardi F (2024). The development of a digital patient navigation tool to increase colorectal cancer screening among federally qualified health center patients: acceptability and usability testing. JMIR Form Res.

[15] Cheng YX, Zhang PL, Hou XY, Wang R, Yan T, Gao CY (2023). “H2H” Nutrition management for colorectal cancer chemotherapy patients based on an internet platform. Journal of Nursing Science May.

[16] Liu W, Cao J, Yi J (2024). The application effect of “Internet” continuity care pathway in patients with chronic diseases. Practical Clinical Medicine.

[17] Im C, Hebdon MCT, Clifford NE, Phillips C (2024). HSR24-149: mobile health (mHealth) interventions for colorectal cancer (CRC) survivors: a systematic review. J Natl Compr Canc Netw.

[18] Briggs LG, Parke SC, Beck KL (2025). Prehabilitative/rehabilitative exercise, nutrition, and psychological support for bladder cancer: a scoping review of randomized clinical trials. Cancer.

[19] Su CC, Guo SE, Kuo YW (2024). Effects of internet-based digital health interventions on the physical activity and quality of life of colorectal cancer survivors: a systematic review and meta-analysis. Support Care Cancer.

[20] Knobloch K, Yoon U, Vogt PM (2011). Preferred reporting items for systematic reviews and meta-analyses (PRISMA) statement and publication bias. J Craniomaxillofac Surg.

[21] Rethlefsen ML, Kirtley S, Waffenschmidt S (2021). PRISMA-S: an extension to the PRISMA Statement for Reporting Literature Searches in Systematic Reviews. Syst Rev.

[22] Higgins JPT, Altman DG, Gøtzsche PC (2011). The Cochrane collaboration’s tool for assessing risk of bias in randomised trials. BMJ.

[23] IntHout J, Ioannidis JPA, Borm GF (2014). The Hartung-Knapp-Sidik-Jonkman method for random effects meta-analysis is straightforward and considerably outperforms the standard DerSimonian-Laird method. BMC Med Res Methodol.

[24] Cohen J (1992). A power primer. Psychol Bull.

[25] Borenstein M, Higgins JPT, Hedges LV, Rothstein HR (2017). Basics of meta-analysis: I^2^ is not an absolute measure of heterogeneity. Res Synth Methods.

[26] Lau J, Ioannidis JPA, Terrin N, Schmid CH, Olkin I (2006). The case of the misleading funnel plot. BMJ.

[27] Furuya-Kanamori L, Rousou X, Kostoulas P, Doi SAR (2025). Examining and interpreting doi plot asymmetry in meta-analyses of randomized controlled trials. J Evid Based Med.

[28] Afonso J, Ramirez-Campillo R, Clemente FM, Büttner FC, Andrade R (2024). The perils of misinterpreting and misusing “Publication Bias” in meta-analyses: an education review on funnel plot-based methods. Sports Med.

[29] Kim YI, Park IJ, Ro JS (2025). A randomized controlled trial of a digital lifestyle intervention involving postoperative patients with colorectal cancer. NPJ Digit Med.

[30] Hwang YJ, Kim IY, Hur HK, Lee JY, Park S (2025). The effects of an app-based physical activity program on colorectal cancer patients undergoing chemotherapy: a randomized controlled trial. Cancer Nurs.

[31] Nunthanawanich P, Wichansawakun S, Luangjinda C, Hudthagosol C (2024). Effectiveness of web applications on improving nutritional status of patients with colorectal cancer. Nutrients.

[32] Rocamora González C, Rodríguez Vega B, Torrijos Zarcero M (2022). Mindfulness based intervention through mobile app for colorectal cancer people awaiting surgery: a randomized clinical trial. Cirugía Española (English Edition).

[33] Chan H, Van Loon K, Kenfield SA (2022). Quality of life of colorectal cancer survivors participating in a pilot randomized controlled trial of physical activity trackers and daily text messages. Support Care Cancer.

[34] Huang Q, Zhuang Y, Ye X (2021). The effect of online training-based continuous nursing care for rectal cancer-patients undergoing permanent colostomy. Am J Transl Res.

[35] Vos JAM, Duineveld LAM, Wieldraaijer T (2021). Effect of general practitioner-led versus surgeon-led colon cancer survivorship CARE, with or without eHealth support, on quality of life (I CARE): an interim analysis of 1-year results of a randomised, controlled trial. Lancet Oncol.

[36] Qu L, Zhou M, Yu Y, Li K (2022). Effects of nutritious meal combined with online publicity and education on postoperative nutrition and psychological state in patients with low rectal cancer after colostomy. Comput Math Methods Med.

[37] Xu L, Zhou MZ (2023). Effect of internet multiple linkage mode-based extended care combined with in-hospital comfort care on colorectal cancer patients undergoing colostomy. World J Gastrointest Surg.

[38] Kim BY, Park KJ, Ryoo SB (2018). Effects of a mobile educational program for colorectal cancer patients undergoing the enhanced recovery after surgery. Open Nurs J.

[39] Nguyen HT, Tsai HH, Huynh HTP (2025). Effectiveness of web-based education program on knowledge, coping, burden, and quality of life among colorectal cancer caregivers in Vietnam: a quasi-experimental study. BMC Nurs.

[40] Sterne JAC, Sutton AJ, Ioannidis JPA (2011). Recommendations for examining and interpreting funnel plot asymmetry in meta-analyses of randomised controlled trials. BMJ.

[41] Molnar F, Man-Son-Hing M, Byszewski A, Azid N (2000). Interpreting the results of small trials. CMAJ.

[42] Qiao J, Zhao Y, Lu Y, Li Q, Dong HJ (2024). Assessing the impact of educational eHealth and mHealth interventions on health outcomes in continuity of care for enterostomy patients: a meta-analysis. Eur J Oncol Nurs.

[43] Hao J, Xu Y, Li H (2023). The value of applying a continuous nursing model based on virtual platforms for patients with colostomy or ileostomy. Adv Skin Wound Care.

[44] Liu Y, Xu CP, Yang GD (2025). Research progress on the application of digital health intervention in patients with chronic wounds. Journal of Nurses Training.

[45] Wang J, Huang Y, Zheng X (2025). Effect of nutritional intervention on chemotherapy tolerance and quality of life in patients with colorectal cancer undergoing postoperative chemotherapy: a randomized controlled study. Nutr Cancer.

[46] Kruse CS, Betancourt JA, Gonzales M, Dickerson K, Neer M (2022). Leveraging mobile health to manage mental health/behavioral health disorders: systematic literature review. JMIR Ment Health.

[47] Ibrahim ME, M Osman HM, Mubarak Osman AME (2025). Comparing telemedicine and in‑person psychological interventions for anxiety: a systematic review. Cureus.

[48] Hepburn J, Williams L, McCann L (2025). Barriers to and facilitators of digital health technology adoption among older adults with chronic diseases: updated systematic review. JMIR Aging.

[49] Zhou C, Ai Y, Wang S (2024). Barriers and facilitators to participation in electronic health interventions in older adults with cognitive impairment: an umbrella review. BMC Geriatr.

[50] Gulliford M, Alageel S (2019). Digital health intervention at older ages. Lancet Digit Health.

[51] Di Pumpo M, Miatton A, Riccardi MT (2024). Physical activity for the elderly: systematic review and synthesis of digital health interventions. Eur J Public Health.

[52] Kaltenböck J, Hölzle P, Grote V (2023). Effects of psycho-oncologic interventions on emotional distress and quality of life in adult patients with cancer: systematic review and meta-analysis. J Clin Oncol.

[53] Ware JE, Snow KK, Kosinski M (1993). SF-36 health survey.

[54] Fayers P, Bottomley A (2002). Quality of life research within the EORTC—the EORTC QLQ-C30. Eur J Cancer.

[55] Ilic-Zivojinovic J, Krdzic I, Jovanovic A (2022). Transcultural adaptation and validation of the Serbian version of the colorectal-specific quality of life questionnaire FACT-C. PLoS One.

[56] The Whoqol Group (1998). Development of the World Health Organization WHOQOL-BREF quality of life assessment. Psychol Med.

[57] Skibsted CV, Jensen BT, Juul T, Kristensen HØ (2022). Patient reported outcome measures assessing quality of life in patients with an intestinal stoma: a systematic review. Colorectal Dis.

[58] Pilz MJ, Thurner AMM, Storz LM, Krepper D, Giesinger JM (2025). The current use and application of thresholds for clinical importance of the EORTC QLQ-C30, the EORTC CAT core and the EORTC QLQ-C15-PAL- a systematic scoping review. Health Qual Life Outcomes.

[59] Jung WB (2024). Beyond survival: a comprehensive review of quality of life in rectal cancer patients. Ann Coloproctol.

