# Supplementary material for: Effect of Digital Health Interventions on the Nutritional Status and Quality of Life of Patients With Colorectal Cancer: Systematic Review and Meta-Analysis of Randomized Controlled Trials
Source: J Med Internet Res. 2026 Aug 18;28:e87866. doi: 10.2196/87866 (PMC13486467; doi:10.2196/87866)
Supplement: Supplementary file 2 [file jmir-v28-e87866-s003.docx]

**Table S1.** Characteristics of included randomized controlled trials,patients undergoing digital health intervention,sample sizes per arm,participant demographics,intervention duration,primary outcomes and follow-up timepoints,and countrynd year of trial conduct.

| **Studies** | **Countries** | **Study design** | **Study type** | **Participants, n** | **Age (y), mean (SD)** | **Intervention duration (weeks), n** | **Intervention and control arm** | **Involvement of health care professionals** | **Measures to ensure intervention adherence** | **Outcomes** | **Measurement scale** |
| --- | --- | --- | --- | --- | --- | --- | --- | --- | --- | --- | --- |
| Kim et al [29], 2025 | Korea | Clinical outcome | RCT^a^ | E ^b^ : 79; C ^c^ : 80 | E:68 people over the age of 40;  C:63 people over the age of 40 | 24 | Intervention:lifestyle intervention based mHealth application; control:routine outpatient  rehabilitation | Coach and nutritionist | Send SMS  text message | Qol | SF-36^d^ |
| Hwang et al [30], 2025 | Korea | Clinical outcome | RCT | E:17  C:17 | E:62.18±7.18  C:63.29±7.10 | 6 | Intervention: an app-based physical activity program; control:standard booklet education | Nurse | Follow-up,  in-person  assessments | Qol | EORTC QLQ-C30^e^ |
| Nunthanawanich et al [31], 2024 | Thailand | Clinical outcome | RCT | E:13  C:15 | E:63.77±2.26  C:61.07±2.12 | 3 | Intervention: nutrition  educational prototype based on smartphone web applications ; control:standard hospital leaflets | Oncologist | Not stated | NS^f^ | ALB^g^ |
| Gonza´lez et al [32], 2022 | Spain | Clinical outcome | RCT | E:39  C:43 | E:63.70  C:66.40 | 4 | Intervention: a mindfulness training through a mobile application ; control:usual care | Research  assistant | Not stated | Qol | WHOQOL-BREF^h^ |
| Chan et al [33], 2022 | USA | Pilot  study | RCT | E:20  C:21 | E:55.6±12.3  C:54.4±10.6 | 12 | Intervention: received a Fitbit Flex™,daily text messages, and print education material;  control: only received print education material | Not stated | Fitbit wear and send daily  text message | Qol | FACT-C^i^ |
| Huanget al [34], 2021 | China | Clinical outcome | RCT | E:62  C:57 | E:45.82±7.02  C:46.18±8.12 | 24 | Intervention:online training-based continuous  nursing care; control:routine nursing  care | A deputy chief physician or nurse | Routine telephone follow-up | Qol | SF-36 |
| Julienet al [35], 2021 | Netherlands | Clinical outcome | RCT | E:141  C:148 | E:67.5（63~74）  C:69（63~75） | 12 | Intervention:Care based on eHealth app; control:usual care. | General practitioners and surgeons | Send daily  text message | Qol | EORTC QLQ-C30 |
| Studies | Countries | Study design | Study type | Participants, n | Age (y), mean (SD) | Intervention duration (weeks), n | Intervention and control arm | Involvement of health care professionals | Measures to ensure intervention adherence | Outcomes | Measurement scale |
| Qu et al [36], 2022 | China | Clinical outcome | RCT | E:44  C:44 | E:65.59±9.28  C:65.91±9.73 | 48 | Intervention:nutritious meal combined with online publicity and  education; control:conventional care | The primary nurses and the doctor | By  phone to inform | NS  Qol | ALB  WHOQOL-BREF |
| Li et al [37], 2023 | China | Clinical outcome | RCT | E:62  C:63 | E:47.82±5.42  C:47.36 ± 4.59 | 12 | Intervention: internet multiple linkage mode-based extended care combined with in-hospital comfort care;control:usual care | 2 attending Doctors and 13 nurses | WeChat message notification | Qol | COH-QOL-OQ^j^ |
| Kim et al [38], 2018 | Korea | Clinical outcome | RCT | E:59  C:59 | E:58±10.13  C:63±9.77 | 2 | Intervention: a mobile mobile web-based educational program;  control:conventional care | Not stated | Sending text messages and making follow-up calls | Qol | FACT-C |
| Nguyen et al [39], 2025 | Vietnam | Clinical outcome | RCT | E:62  C:65 | E:41.79±12.20  C:37.7±9.8 | 12 | Intervention:a web-based  education program;  control: regular care | Five  oncology nurses, five specialist physicians, and 10 caregivers | Not stated | Qol | COH-QOL-OQ |

^a^RCT: randomized controlled trial.

^b^E: experimental group.

^c^C: control group

^d^SF-36: 36-Item Short Form Health Survey

^e^EORTC QLQ-C30: European Organization for Research and Treatment of Cancer—Quality of Life Core 30

^f^NS: Nutritional Status

^g^ALB: Albumin

^h^WHOQOL-BREF: The World Health Organization Quality of Life -BREF

^i^FACT-C: Functional Assessment of Cancer Therapy–Colorectal

^j^COH-QOL-OQ: Hope-Quality of life-Ostomy Questionnaire

**Table S2.** Digital health tools characteristics.

| Studies | Digital health tools name | General objective | Specific objectives | Devices | Main features of the digital health tools |
| --- | --- | --- | --- | --- | --- |
| Kim et al [29], 2025 | The Second Doctor© application | Rehabilitation | Improve quality of life | A tablet computer and a smart band | Offer one-on-one consultations with human coaches, including nutritionists, and patients can automatically record their daily step count, consumed calories, heartbeat, and sleep patterns using a smart band |
| Hwang et al [30], 2025 | App-Based Physical Activity Program | Self-management | Improve exercise adherence and reduce peripheral neuropathy symptoms | A smartphone | Set individual exercise goals with the participants on the day of discharge;  Follow the exercises prescribed by the application downloaded on mobile phone;  Provide the function of answering questions. |
| Nunthanawanich et al [31], 2024 | NEPSA^a^ | Self-management | Improve nutrition and democratize nutritional information | A smartphone or tablet with screen | Provide easy access to scientific nutritional information;  Six modules for providing nutritional consultation: nutritional news, weekly self-body weight recorder, food group selection, fruit and vegetable washing techniques, fruit and vegetable washing techniques, and medical food selection |
| Gonza´lez et al [32], 2022 | Calm in the operating room | Rehabilitation | Reduce anxious-depressive symptoms  and improve quality of life | A smartphone | Provide a brief introduction to the calm down programme and a video of the surgical hospital context;  Two training programmes are shown: the long programme (surgery scheduled in 15 days or a month) and the short programme (surgery scheduled in a few hours or days). They can be downloaded free of charge. |
| Chan et al [33], 2022 | Not stated | Self-management | Improve quality of life | A Fitbit Flex™ smart bracelet and accelerometer | Provide a Fitbit Flex, daily text messages, and print education material;  Provide informational and motivational messages on various exercise activities, such as brisk walking, jogging, and resistance training. |
| Huanget al [34], 2021 | Not stated | Rehabilitation | Improve life quality and self-care ability | A smartphone | Establish WeChat and QQ groups;  Provide answers to patients’ questions by specified times each week;  Establish an official account to post some knowledge related to health. |
| Julienet al [35], 2021 | Oncokompas | Rehabilitation | Improve quality of life and reduce morbidity | I CARE website | Consist of three components: measure, learn and act;  Patients can look up any topic relevant to their personal situation to obtain personalised feedback. |
| Qu et al [36], 2022 | Not stated | Self-management | Investigate postoperative nutrition  and psychological state | A smartphone | Compose and timely send the information related to ostomy to the WeChat groups in the form of text, picture, audio, and video;  Check the chat contents in the group and promptly answer the inquiries from patients and their families regarding the illness every day. |
| Li et al [37], 2023 | Colorectal microplatform | Self-management | Improve self-efficacy, bad mood,cancer-related fatigue and life quality | A tablet computer and a mobile phone | The applet included the medical side and the patient side. The medical side had three sections: information verification;health knowledge; and physician-patient interaction. The patient side included the patient’s personal data, information platform and interaction. |
| Kim et al [38], 2018 | Mobile-Based Educational Program | Self-management | Improve the quality of life and self-efficacy and reduce anxiety and depression | A mobile phone | Includes eight aspects: objective, credibility, interaction,update, accessibility,function, design, and security. |
| Nguyen et al [39], 2025 | Web-based education program | Self-management | Manage caregiving  duties, enhance coping abilities, and improving quality of life | Mobiles, tablets, and computers | Access the website directly through the provided link and searched for information based on their needs during the study period;  Monitor, provide feedback, and support individual participants using several methods. |

^a^Nutrition Educational Prototype based on Smartphone Web Applications (NEPSA)
