# Supplementary material for: Effect of Digital Health Interventions on the Nutritional Status and Quality of Life of Patients With Colorectal Cancer: Systematic Review and Meta-Analysis of Randomized Controlled Trials
Source: J Med Internet Res. 2026 Aug 18;28:e87866. doi: 10.2196/87866 (PMC13486467; doi:10.2196/87866)
Supplement: Supplementary file 3 [file jmir-v28-e87866-s002.docx]

Leave-One-Out Sensitivity for QoL
