# Supplementary material for: Effect of Digital Health Interventions on the Nutritional Status and Quality of Life of Patients With Colorectal Cancer: Systematic Review and Meta-Analysis of Randomized Controlled Trials
Source: J Med Internet Res. 2026 Aug 18;28:e87866. doi: 10.2196/87866 (PMC13486467; doi:10.2196/87866)
Supplement: Supplementary file 4 [file jmir-v28-e87866-s001.doc]

**Online Resource 1.** Search strategies

Search Updated: March 2nd, 2026

**PubMed**

| **Search** | **Search Terms/Strategy** | **Results** |
| --- | --- | --- |
| #1 | [Title/Abstract]: ((((("Colorectal Neoplasms"[Mesh]) OR ((((((((((((((((Colorectal Neoplasms) OR Colorectal Neoplasm) OR Neoplasm, Colorectal) OR Colorectal Tumors) OR Colorectal Tumor) OR Tumor, Colorectal) OR Tumors, Colorectal) OR Neoplasms, Colorectal) OR Colorectal Cancer) OR Cancer, Colorectal) OR Cancers, Colorectal) OR Colorectal Cancers) OR Colorectal Carcinoma) OR Carcinoma, Colorecta) OR Carcinomas, Colorectal) OR Colorectal Carcinomas)) OR ("Colonic Neoplasms"[Mesh])) OR ((((((((((((((((((((((Colonic Neoplasms) OR Colonic Neoplasm) OR Neoplasm, Colonic) OR Colon Neoplasms) OR Colon Neoplasm) OR Neoplasm, Colon) OR Neoplasms, Colon) OR Neoplasms, Colonic) OR Cancer of Colon) OR Colon Cancers) OR Cancer of the Colon) OR Colonic Cancer) OR Cancer, Colonic) OR Cancers, Colonic) OR Colonic Cancers) OR Colon Cancer) OR Cancer, Colon) OR Cancers, Colon) OR Colon Adenocarcinoma) OR Adenocarcinoma, Colon) OR Adenocarcinomas, Colon) OR Colon Adenocarcinomas)) OR ("Rectal Neoplasms"[Mesh])) OR ((((((((((((((((((Rectal Neoplasms) OR Neoplasm, Rectal) OR Rectal Neoplasm) OR Neoplasms, Rectal) OR Rectum Neoplasms) OR Neoplasm, Rectum) OR Rectum Neoplasm) OR Rectal Tumors) OR Rectal Tumor) OR Tumor, Rectal) OR Cancer of Rectum) OR Rectum Cancers) OR Cancer of the Rectum) OR Rectal Cancer) OR Cancer, Rectal) OR Rectal Cancers) OR Rectum Cancer) OR Cancer, Rectum) | 411736 |
| #2 | ((((((((((("Digital Health"[Mesh]) OR (((((Health, Digital) OR (Digital Health Technology)) OR (Digital Health Technologies)) OR (Health Technologies, Digital)) OR (Health Technology, Digital))) OR ("Telemedicine"[Mesh])) OR (((((((((((((((((Virtual Medicine) OR (Medicine, Virtual)) OR (Tele-Referral)) OR (Tele Referral)) OR (Tele-Referrals)) OR (Mobile Health)) OR (Health, Mobile)) OR (mHealth)) OR (Telehealth)) OR (eHealth)) OR (Tele-Intensive Care)) OR (Tele Intensive Care)) OR (Tele-ICU)) OR (Tele ICU)) OR (Telecare)) OR (Tele-Care)) OR (Tele Care))) OR ("Remote Patient Monitoring"[Mesh])) OR ((Monitoring, Remote Patient) OR (Patient Monitoring, Remote))) OR ("Smartphone"[Mesh])) OR ((((Smartphones) OR (Smart Phone)) OR (Phones, Smart)) OR (Smart Phones))) OR ("Mobile Applications"[Mesh])) OR (((((((((((((((((((((((((((Application, Mobile) OR (Applications, Mobile)) OR (Mobile Application)) OR (Mobile Apps)) OR (App, Mobile)) OR (Apps, Mobile)) OR (Mobile App)) OR (Portable Electronic Apps)) OR (App, Portable Electronic)) OR (Electronic App, Portable)) OR (Portable Electronic App)) OR (Portable Electronic Applications)) OR (Application, Portable Electronic)) OR (Electronic Application, Portable)) OR (Portable Electronic Application)) OR (Portable Software Apps)) OR (App, Portable Software)) OR (Portable Software App)) OR (Software App, Portable)) OR (Portable Software Applications)) OR (Application, Portable Software)) OR (Portable Software Application)) OR (Software Application, Portable)) OR (Smartphone Apps)) OR (App, Smartphone)) OR (Apps, Smartphone)) OR (Smartphone App))) OR ("Internet-Based Intervention"[Mesh])) OR (((((((((((((((((Internet Based Intervention) OR (Internet-Based Interventions)) OR (Intervention, Internet-Based)) OR (Interventions, Internet-Based)) OR (Internet Intervention)) OR (Internet Interventions)) OR (Intervention, Internet)) OR (Interventions, Internet)) OR (Web-based Intervention)) OR (Interventions, Web-based)) OR (Intervention, Web-based)) OR (Web based Intervention)) OR (Web-based Interventions)) OR (Online Intervention)) OR (Intervention, Online)) OR (Interventions, Online)) OR (Online Interventions)) | 364988 |
| #3 | ((((Nutritional Status [Mesh]) OR (((Status, Nutritional) OR (Nutrition Status)) OR (Status, Nutrition))) OR (Quality of Life [Mesh])) OR ((((Life Quality) OR (Health-Related Quality Of Life)) OR (Health Related Quality Of Life)) OR (HRQOL))) | 809199 |
| #4 | #1 AND #2 AND #3 | 357 |

**Embase**

| **Search** | **Search Terms/Strategy** | **Results** |
| --- | --- | --- |
| #1 | 'colorectal tumor'/exp | 542167 |
| #2 | 'colorectal neoplasia':ti,ab,kw OR 'colorectal neoplasm':ti,ab,kw OR 'colorectal neoplasms':ti,ab,kw OR 'colorectal tumorigenesis':ti,ab,kw OR 'colorectal tumour':ti,ab,kw OR 'neoplastic colorectal':ti,ab,kw OR 'tumor,colorectal':ti,ab,kw OR 'tumour, colorectal':ti,ab,kw OR 'colorectal tumor':ti,ab,kw | 20229 |
| #3 | 'colon tumor'/exp | 199315 |
| #4 | 'colon mass tumor':ti,ab,kw OR 'colon neoplasia':ti,ab,kw OR 'colon neoplasm':ti,ab,kw OR 'colon tumorigenesis':ti,ab,kw OR 'colon tumour':ti,ab,kw OR 'colon villous tumor':ti,ab,kw OR 'colon villous tumour':ti,ab,kw OR 'colonic mass tumor':ti,ab,kw OR 'colonic masses tumor':ti,ab,kw OR 'colonic neoplasia':ti,ab,kw OR 'colonic neoplasm':ti,ab,kw OR 'colonic neoplasms':ti,ab,kw OR 'colonic tumor':ti,ab,kw OR 'colonic tumorigenesis':ti,ab,kw OR 'colonic tumour':ti,ab,kw OR 'mesocolon tumor':ti,ab,kw OR 'mesocolon tumour':ti,ab,kw OR 'neoplasia in the colon':ti,ab,kw OR 'neoplasia of the colon':ti,ab,kw OR 'neoplastic colon':ti,ab,kw OR 'neoplastic colonic':ti,ab,kw OR 'tumor in the colon':ti,ab,kw OR 'tumor of the colon':ti,ab,kw OR 'tumorigenesis in colon':ti,ab,kw OR 'colon tumor':ti,ab,kw | 10486 |
| #5 | 'rectum tumor'/exp | 94860 |
| #6 | 'mass, rectum tumor':ti,ab,kw OR 'neoplasm of the rectum':ti,ab,kw OR 'neoplasma recti':ti,ab,kw OR 'pararectal tumor':ti,ab,kw OR 'pararectal tumour':ti,ab,kw OR 'rectal mass tumor':ti,ab,kw OR 'rectal neoplasia':ti,ab,kw OR 'rectal neoplasm':ti,ab,kw OR 'rectal neoplasms':ti,ab,kw OR 'rectal tumor':ti,ab,kw OR 'rectal tumour':ti,ab,kw OR 'rectum mass tumor':ti,ab,kw OR 'rectum neoplasia':ti,ab,kw OR 'rectum neoplasm':ti,ab,kw OR 'rectum tumour':ti,ab,kw OR 'retrorectal tumor':ti,ab,kw OR 'retrorectal tumour':ti,ab,kw OR 'tumor of the rectum':ti,ab,kw OR 'tumor recti':ti,ab,kw OR 'tumour of the rectum':ti,ab,kw OR 'tumour recti':ti,ab,kw OR 'rectum tumor':ti,ab,kw | 3279 |
| #7 | #1 OR #2 OR #3 OR #4 OR #5 OR #6 | 544578 |
| #8 | 'nutritional status'/exp | 98802 |
| #9 | 'nutrition state':ti,ab,kw OR 'nutrition status':ti,ab,kw OR 'nutritional state':ti,ab,kw OR 'nutritive state':ti,ab,kw OR 'nutritive status':ti,ab,kw OR 'nutritional status':ti,ab,kw | 78893 |
| #10 | #8 OR #9 | 119250 |
| #11 | 'quality of life'/exp | 829260 |
| #12 | 'health related quality of life':ti,ab,kw OR 'hrql':ti,ab,kw OR 'life quality':ti,ab,kw OR 'quality of life':ti,ab,kw | 759206 |
| #13 | #11 OR #12 | 997704 |
| #14 | 'digital health'/exp | 5884 |
| #15 | 'telemedicine'/exp | 90131 |
| #16 | 'tele medicine':ti,ab,kw OR 'virtual medicine':ti,ab,kw OR 'telemedicine':ti,ab,kw | 42307 |
| #17 | 'telemonitoring'/exp | 8093 |
| #18 | 'distant monitoring patient':ti,ab,kw OR 'distant patient monitoring':ti,ab,kw OR 'remote distance patient monitoring':ti,ab,kw OR 'remote monitoring patient':ti,ab,kw OR 'remote patient monitoring':ti,ab,kw OR 'remote patient surveillance':ti,ab,kw OR 'tele monitoring':ti,ab,kw OR 'tele surveillance':ti,ab,kw OR 'telesurveillance':ti,ab,kw OR 'telemonitoring':ti,ab,kw | 11633 |
| #19 | 'smartphone'/exp | 40129 |
| #20 | 'smart phone':ti,ab,kw OR 'smartphones':ti,ab,kwOR 'smartphone':ti,ab,kw | 45094 |
| #21 | 'mobile application'/exp | 36685 |
| #22 | 'mobile app':ti,ab,kw OR 'mobile applications':ti,ab,kw OR 'mobile apps':ti,ab,kw OR 'portable software app':ti,ab,kw OR 'portable software application':ti,ab,kw OR 'portable software applications':ti,ab,kw OR 'portable software apps':ti,ab,kw OR 'tablet application':ti,ab,kw OR 'mobile application':ti,ab,kw | 19437 |
| #23 | 'web-based intervention'/exp | 5536 |
| #24 | 'internet-based intervention':ti,ab,kw OR 'internet-intervention':ti,ab,kw OR 'online-based intervention':ti,ab,kw OR 'online-intervention':ti,ab,kw OR 'web intervention':ti,ab,kw OR 'web-based intervention':ti,ab,kw | 5446 |
| #25 | #14 OR #15 OR #16 OR #17 OR #18 OR #19 OR #20 OR #21 OR #22 OR #23 OR #24 | 184778 |
| #26 | #7 AND #10 AND #25 | 14 |
| #27 | #7 AND #13 AND #25 | 239 |
| #28 | #26 OR #27 | 249 |

**Cochrane Library (CENTRAL)**

| **Search** | **Search Terms/Strategy** | **Results** |
| --- | --- | --- |
| #1 | (Colorectal Neoplasms):ti,ab,kw OR (Colorectal Neoplasm):ti,ab,kw OR (Neoplasms, Colorectal):ti,ab,kw OR (Neoplasm, Colorectal):ti,ab,kw OR (Colorectal Tumors):ti,ab,kw | 13188 |
| #2 | (Tumors,Colorectal):ti,ab,kw OR (Colorectal Tumor):ti,ab,kw OR (Tumor, Colorectal):ti,ab,kwOR (Colorectal Cancers):ti,ab,kw OR (Cancer, Colorectal):ti,ab,kw | 20619 |
| #3 | (Cancers, Colorectal):ti,ab,kw OR (Colorectal Carcinoma):ti,ab,kw OR (Carcinomas, Colorectal):ti,ab,kw OR (Colorectal Carcinomas):ti,ab,kw OR (Colorectal Cancer):ti,ab,kw | 20504 |
| #4 | Carcinoma, Colorectal | 2671 |
| #5 | (Colonic Neoplasms):ti,ab,kw OR (Colonic Neoplasm):ti,ab,kw OR (Colon Neoplasms):ti,ab,kw OR (Colon Neoplasm):ti,ab,kw | 5700 |
| #6 | (Rectal Neoplasms):ti,ab,kw OR (Cancer, Rectal):ti,ab,kw OR (Cancer, Rectum):ti,ab,kw OR (Rectal Cancers):ti,ab,kw OR (Cancer of the Rectum):ti,ab,kw | 9208 |
| #7 | (Rectal Cancer):ti,ab,kw OR (Rectum Cancers):ti,ab,kw OR (Cancer of Rectum):ti,ab,kw OR (Rectum Cancer):ti,ab,kw OR (Neoplasm, Rectum):ti,ab,kw | 8747 |
| #8 | (Rectal Tumor):ti,ab,kw OR (Neoplasm, Rectal):ti,ab,kw OR (Rectal Tumors):ti,ab,kw OR (Neoplasms, Rectal):ti,ab,kw OR (Tumor, Rectal):ti,ab,kw | 5822 |
| #9 | (Rectal Neoplasm):ti,ab,kw OR (Rectum Neoplasm):ti,ab,kw OR (Rectum Neoplasms):ti,ab,kw | 3484 |
| #10 | （#1）OR（#2）OR（#3）OR（#4）OR（#5）OR（#6）OR（#7）OR（#8）OR（#9） | 112264 |
| #11 | Nutritional Status):ti,ab,kw OR (Nutrition Status):ti,ab,kw | 15661 |
| #12 | (Quality of Life):ti,ab,kw OR (HRQOL):ti,ab,kw OR (Health Related Quality Of Life):ti,ab,kw OR (Health-Related Quality Of Life):ti,ab,kw OR (Life Quality):ti,ab,kw | 189138 |
| #13 | (Digital Health):ti,ab,kw OR (Health Technologies, Digital):ti,ab,kw OR (Digital Health Technology):ti,ab,kw OR (Health Technology, Digital):ti,ab,kw OR (Digital Health Technologies):ti,ab,kw | 8912 |
| #14 | (Telemedicine):ti,ab,kw OR (Medicine, Virtual):ti,ab,kw OR (Tele-Referrals):ti,ab,kw OR (Tele Referral):ti,ab,kw OR (Virtual Medicine):ti,ab,kw | 8060 |
| #15 | (Tele-Referral;):ti,ab,kw OR (Tele-Intensive Care):ti,ab,kw OR (Tele ICU):ti,ab,kw OR (Tele Intensive Care):ti,ab,kw OR (Tele-ICU):ti,ab,kw | 91 |
| #16 | (Telecare):ti,ab,kw OR (Tele-Care):ti,ab,kw OR (Tele Care):ti,ab,kw OR (Health, Mobile):ti,ab,kw OR (mHealth):ti,ab,kw | 13909 |
| #17 | (Mobile Health):ti,ab,kw OR (Telehealth):ti,ab,kw OR (eHealth):ti,ab,kw | 17222 |
| #18 | (Remote Patient Monitoring):ti,ab,kw OR (Patient Monitoring, Remote):ti,ab,kw OR (Monitoring, Remote Patient):ti,ab,kw OR (Smartphone):ti,ab,kw OR (Smartphones):ti,ab,kw | 11389 |
| #19 | (Mobile Applications):ti,ab,kw OR (Mobile Application):ti,ab,kw OR (Apps, Mobile):ti,ab,kw OR (Mobile Apps):ti,ab,kw OR (App, Mobile):ti,ab,kw | 10413 |
| #20 | (Application, Mobile):ti,ab,kw OR (Applications, Mobile):ti,ab,kw OR (Mobile App):ti,ab,kw OR (Smartphone Apps):ti,ab,kw OR (Apps, Smartphone):ti,ab,kw | 10631 |
| #21 | (App, Smartphone):ti,ab,kw OR (Smartphone App):ti,ab,kw OR (Portable Electronic Application):ti,ab,kw OR (Portable Electronic Apps):ti,ab,kw OR (Electronic App, Portable):ti,ab,kw | 4055 |
| #22 | (App, Portable Electronic):ti,ab,kw OR (Portable Electronic Applications):ti,ab,kw OR (Application, Portable Electronic):ti,ab,kw OR (Electronic Application, Portable):ti,ab,kw OR (Portable Electronic App):ti,ab,kw | 47 |
| #23 | (Software Application, Portable):ti,ab,kw OR (Application, Portable Software):ti,ab,kw OR (Portable Software Applications):ti,ab,kw OR (Software App, Portable):ti,ab,kw OR (Portable Software Apps):ti,ab,kw | 79 |
| #24 | (App, Portable Software):ti,ab,kw OR (Portable Software App):ti,ab,kw OR (Portable Software Application):ti,ab,kw | 68 |
| #25 | (Internet-Based Intervention):ti,ab,kw OR (Internet-Based Interventions):ti,ab,kw OR (Intervention, Web-based):ti,ab,kw OR (Internet Based Intervention):ti,ab,kw OR (Intervention, Internet):ti,ab,kw | 17292 |
| #26 | (Interventions,Internet-Based):ti,ab,kw OR (Web based Intervention):ti,ab,kw OR (Interventions, Internet):ti,ab,kw OR (Interventions, Web-based):ti,ab,kw OR (Intervention, Internet-Based):ti,ab,kw | 15261 |
| #27 | (Online Interventions):ti,ab,kw OR (Online Intervention):ti,ab,kw OR (Internet Intervention):ti,ab,kw OR (Internet Interventions):ti,ab,kw OR (Web-based Intervention):ti,ab,kw | 31565 |
| #28 | (Web-based Interventions):ti,ab,kw OR (Interventions, Online):ti,ab,kw OR (Intervention, Online):ti,ab,kw | 22128 |
| #29 | （#13）OR（#14）OR（#15）OR（#16）OR（#17）OR（#18）OR（#19）OR（#20）OR（#21）OR（#22）OR（#23）OR（#24）OR（#25）OR（#26）OR（#27）OR（#28） | 65446 |
| #30 | #10 AND #11 AND #29 | 13 |
| #31 | #10 AND #12 AND #29 | 19 |
| #32 | #30 OR #31 | 32 |

**Web of Science**

| **Search** | **Search Terms/Strategy** | **Results** |
| --- | --- | --- |
| #1 | TS= (Colorectal Neoplasms OR Colorectal Neoplasm OR Neoplasm, Colorectal OR Colorectal Tumors OR Colorectal Tumor OR Tumor, Colorectal OR Tumors, Colorectal OR Neoplasms, Colorectal OR Colorectal Cancer OR Colorectal Cancers OR Cancer, Colorectal OR Cancers, Colorectal OR Colorectal Carcinoma OR Colorectal Carcinomas OR Carcinoma, Colorecta OR Carcinomas, Colorecta OR Colonic Neoplasms OR Colonic Neoplasm OR Neoplasm, Colonic OR Colon Neoplasms OR Colon Neoplasm OR Neoplasm, Colon OR Neoplasms, Colon OR Neoplasms, Colonic OR Cancer of Colon OR Colon Cancers OR Cancer of the Colon OR Colonic Cancer OR Colonic Cancers OR Cancer, Colonic OR Cancers, Colonic OR Colon Cancer OR Cancer, Colon OR Cancers, Colon OR Colon Adenocarcinoma OR Colon Adenocarcinomas OR Adenocarcinoma, Colon OR Adenocarcinomas, Colon OR Rectal Neoplasms OR Neoplasm, Rectal OR Rectal Neoplasm OR Neoplasms, Rectal OR Rectum Neoplasms OR Neoplasm, Rectum OR Rectum Neoplasm OR Rectal Tumors OR Rectal Tumor OR Tumor, Rectal OR Cancer of Rectum OR Rectum Cancers OR Cancer of the Rectum OR Rectal Cancer OR Rectal Cancers OR Cancer, Rectal OR Rectum Cancer OR Cancer, Rectum ) | 662342 |
| #2 | TS= (digital health OR Health, Digital OR Digital Health Technology OR Digital Health Technologies OR Health Technologies, Digital OR Health Technology, Digital OR Telemedicine OR Virtual Medicine OR Medicine, Virtual OR Tele-Referral OR Tele Referral OR Tele-Referrals OR Mobile Health OR Health, Mobile OR mHealth OR Telehealth OR eHealth OR Tele-Intensive Care OR Tele Intensive Care OR Tele-ICU OR Tele ICU OR Telecare OR Tele-Care OR Tele Care OR Remote Patient Monitoring OR Monitoring, Remote Patient OR Patient Monitoring, Remote OR Smartphone OR Smartphones OR Smart Phone OR Phones, Smart OR Smart Phones OR Mobile Applications OR Application, Mobile OR Applications, Mobile OR Mobile Application OR Mobile Apps OR App, Mobile OR Apps, Mobile OR Mobile App OR Portable Electronic Apps OR App, Portable Electronic OR Electronic App, Portable OR Portable Electronic App OR Portable Electronic Applications OR Application, Portable Electronic OR Electronic Application, Portable OR Portable Electronic Application OR Portable Software Apps OR App, Portable Software OR Portable Software App OR Software App, Portable OR Portable Software Applications OR Application, Portable Software OR Portable Software Application OR Software Application, Portable OR Smartphone Apps OR App, Smartphone OR Apps, Smartphone OR Smartphone App OR Internet-based interventions OR Internet Based Intervention OR Internet-Based Interventions OR Intervention, Internet-Based OR Interventions, Internet-Based OR Internet Intervention OR Internet Interventions OR Intervention, Internet OR Interventions, Internet OR Web-based Intervention OR Interventions, Web-based OR Intervention, Web-based OR Web based Intervention OR Web-based Interventions OR Online Intervention OR Intervention, Online OR Interventions, Online OR Online Interventions) | 904415 |
| #3 | TS=(Nutritional status OR Status, Nutritional OR Nutrition Status OR Status, Nutrition OR Quality of Life OR Life Quality OR Health-Related Quality Of Life OR Health Related Quality Of Life OR HRQOL) | 2339538 |
| #4 | #1 AND #2 AND #3 | 970 |
